# Supplementary material for: Bayesian sample size determination for diagnostic accuracy studies
Source: Stat Med. 2022 Apr 10;41(15):2908–22. doi: 10.1002/sim.9393 (PMC9325402; doi:10.1002/sim.9393)
Supplement: Supplementary file 1 — Data S1 Supplementary material [file SIM-41-2908-s002.pdf]

Supporting Information for *Bayesian sample size determination for diagnostic accuracy studies*

Kevin J. Wilson, S. Faye Williamson, A. Joy Allen,
Cameron J. Williams, Thomas P. Hellyer, B. Clare Lendrem

February 21, 2022

A Assurance derivations

(i) The assurance for the *sensitivity* λ, based on a sample size of n_T, and conditional on $n_{T,1}$, is

$$\begin{aligned} A_\lambda(n_T \mid n_{T,1}) &= \int \Pr(\text{Sensitivity achieved} \mid \lambda)\pi(\lambda)d\lambda, \\ &= \int \left[\Pr(n_{1,1} \leq c_1 \mid \lambda) + \Pr(n_{1,1} \geq c_2 \mid \lambda)\right]\pi(\lambda)d\lambda, \end{aligned}$$

where $n_{1,1} \mid n_{T,1}, \lambda \sim \text{Bin}(n_{T,1}, \lambda)$ and $c_1 < c_2$ are critical numbers chosen such that the posterior probability interval will be narrower than w^* when $n_{1,1} \leq c_1$ and $n_{1,1} \geq c_2$. Thus,

$$A_\lambda(n_T \mid n_{T,1}) = \int \left[\sum_{n_{1,1} \in \mathcal{N}} \binom{n_{T,1}}{n_{1,1}} \lambda^{n_{1,1}}(1-\lambda)^{n_{T,1}-n_{1,1}}\right]\pi(\lambda)d\lambda,$$

where $\mathcal{N} = \{n_{1,1} : n_{1,1} \leq c_1 \text{ or } n_{1,1} \geq c_2\}$. Then

$$\begin{aligned} A_\lambda(n_T \mid n_{T,1}) &= \int \sum_{n_{1,1} \in \mathcal{N}} \binom{n_{T,1}}{n_{1,1}} \lambda^{n_{1,1}}(1-\lambda)^{n_{T,1}-n_{1,1}} \times \frac{\Gamma(a_\lambda + b_\lambda)}{\Gamma(a_\lambda)\Gamma(b_\lambda)} \lambda^{a_\lambda - 1}(1-\lambda)^{b_\lambda - 1} d\lambda, \\ &= \frac{\Gamma(a_\lambda + b_\lambda)}{\Gamma(a_\lambda)\Gamma(b_\lambda)} \left[\sum_{n_{1,1} \in \mathcal{N}} \int \binom{n_{T,1}}{n_{1,1}} \lambda^{a_\lambda + n_{1,1} - 1}(1-\lambda)^{b_\lambda + n_{T,1} - n_{1,1} - 1} d\lambda\right], \\ &= \frac{\Gamma(a_\lambda + b_\lambda)}{\Gamma(a_\lambda)\Gamma(b_\lambda)} \sum_{n_{1,1} \in \mathcal{N}} \binom{n_{T,1}}{n_{1,1}} \frac{\Gamma(a_\lambda + n_{1,1})\Gamma(b_\lambda + n_{2,1})}{\Gamma(a_\lambda + b_\lambda + n_{T,1})}. \end{aligned} \quad (1)$$

The *unconditional* assurance for the sensitivity, based on a sample size of n_T, is

$$\begin{aligned} A_\lambda(n_T) &= \sum_{n_{T,1}=0}^{n_T} A_\lambda(n_T \mid n_{T,1}) \times f(n_{T,1}), \\ &= \frac{\Gamma(a_\lambda + b_\lambda)}{\Gamma(a_\lambda)\Gamma(b_\lambda)} \sum_{n_{T,1}=0}^{n_T} \Bigg\{ \sum_{n_{1,1} \in \mathcal{N}} \left[\binom{n_{T,1}}{n_{1,1}} \frac{\Gamma(a_\lambda + n_{1,1})\Gamma(b_\lambda + n_{2,1})}{\Gamma(a_\lambda + b_\lambda + n_{T,1})}\right] \\ &\quad \times \int f(n_{T,1} \mid \rho)\pi(\rho)d\rho \Bigg\}. \end{aligned}$$

Since $n_{T,1} \sim \mathrm{Bin}(n_T, \rho)$ and $\rho \sim \mathrm{Beta}(a_\rho, b_\rho),$ we have

$$\begin{aligned} A_\lambda(n_T) &= \frac{\Gamma(a_\lambda+b_\lambda)}{\Gamma(a_\lambda)\Gamma(b_\lambda)} \sum_{n_{T,1}=0}^{n_T} \left\{ \sum_{n_{1,1}\in\mathcal{N}} \left[\binom{n_{T,1}}{n_{1,1}} \frac{\Gamma(a_\lambda+n_{1,1})\Gamma(b_\lambda+n_{2,1})}{\Gamma(a_\lambda+b_\lambda+n_{T,1})} \right] \right. \\ &\quad \left. \times \int \binom{n_T}{n_{T,1}} \rho^{n_{T,1}}(1-\rho)^{n_{T,2}} \frac{\Gamma(a_\rho+b_\rho)}{\Gamma(a_\rho)\Gamma(b_\rho)} \rho^{a_\rho-1}(1-\rho)^{b_\rho-1} d\rho \right\}, \\ &= \frac{\Gamma(a_\lambda+b_\lambda)}{\Gamma(a_\lambda)\Gamma(b_\lambda)} \sum_{n_{T,1}=0}^{n_T} \left\{ \sum_{n_{1,1}\in\mathcal{N}} \left[\binom{n_{T,1}}{n_{1,1}} \frac{\Gamma(a_\lambda+n_{1,1})\Gamma(b_\lambda+n_{2,1})}{\Gamma(a_\lambda+b_\lambda+n_{T,1})} \right] \right. \\ &\quad \left. \times \binom{n_T}{n_{T,1}} \frac{\Gamma(a_\rho+b_\rho)}{\Gamma(a_\rho)\Gamma(b_\rho)} \frac{\Gamma(a_\rho+n_{T,1})\Gamma(b_\rho+n_{T,2})}{\Gamma(a_\rho+b_\rho+n_T)} \right\}. \end{aligned}$$

(ii) Similarly, the assurance for the *specificity* θ, based on a sample size of n_T, and conditional on $n_{T,2}$, is

$$\begin{aligned} A_\theta(n_T \mid n_{T,2}) &= \int \mathrm{Pr}(\text{Specificity achieved} \mid \theta)\pi(\theta)d\theta, \\ &= \int \left[\mathrm{Pr}(n_{2,2} \le c_1 \mid \theta) + \mathrm{Pr}(n_{2,2} \ge c_2 \mid \theta)\right] \pi(\theta)d\theta, \end{aligned}$$

where $n_{2,2} \mid n_{T,2}, \theta \sim \mathrm{Bin}(n_{T,2}, \theta)$ and $c_1 < c_2$ are critical numbers chosen such that the posterior probability interval will be narrower than w^* when $n_{2,2} \le c_1$ and $n_{2,2} \ge c_2$. Thus,

$$A_\theta(n_T \mid n_{T,2}) = \int \left[\sum_{n_{2,2}\in\mathcal{N}} \binom{n_{T,2}}{n_{2,2}} \theta^{n_{2,2}}(1-\theta)^{n_{T,2}-n_{2,2}} \right] \pi(\theta)d\theta,$$

where $\mathcal{N} = \{n_{2,2} : n_{2,2} \le c_1 \text{ or } n_{2,2} \ge c_2\}$. Then

$$\begin{aligned} A_\theta(n_T \mid n_{T,2}) &= \frac{\Gamma(a_\theta+b_\theta)}{\Gamma(a_\theta)\Gamma(b_\theta)} \sum_{n_{2,2}\in\mathcal{N}} \int \binom{n_{T,2}}{n_{2,2}} \theta^{a_\theta+n_{2,2}-1}(1-\theta)^{b_\theta+n_{T,2}-n_{2,2}-1} d\theta, \\ &= \frac{\Gamma(a_\theta+b_\theta)}{\Gamma(a_\theta)\Gamma(b_\theta)} \sum_{n_{2,2}\in\mathcal{N}} \binom{n_{T,2}}{n_{2,2}} \frac{\Gamma(a_\theta+n_{2,2})\Gamma(b_\theta+n_{1,2})}{\Gamma(a_\theta+b_\theta+n_{T,2})}. \end{aligned} \qquad (2)$$

The *unconditional* assurance for the specificity, based on a sample size of n_T, is

$$\begin{aligned} A_\theta(n_T) &= \sum_{n_{T,2}=0}^{n_T} A_\theta(n_T \mid n_{T,2}) \times f(n_{T,2}), \\ &= \frac{\Gamma(a_\theta+b_\theta)}{\Gamma(a_\theta)\Gamma(b_\theta)} \sum_{n_{T,2}=0}^{n_T} \left\{ \sum_{n_{2,2}\in\mathcal{N}} \left[\binom{n_{T,2}}{n_{2,2}} \frac{\Gamma(a_\theta+n_{2,2})\Gamma(b_\theta+n_{1,2})}{\Gamma(a_\theta+b_\theta+n_{T,2})} \right] \right. \\ &\quad \left. \times \int f(n_{T,2} \mid \rho)\pi(\rho)d\rho \right\}, \\ &= \frac{\Gamma(a_\theta+b_\theta)}{\Gamma(a_\theta)\Gamma(b_\theta)} \sum_{n_{T,2}=0}^{n_T} \left\{ \sum_{n_{2,2}\in\mathcal{N}} \left[\binom{n_{T,2}}{n_{2,2}} \frac{\Gamma(a_\theta+n_{2,2})\Gamma(b_\theta+n_{1,2})}{\Gamma(a_\theta+b_\theta+n_{T,2})} \right] \right. \\ &\quad \left. \times \int \binom{n_T}{n_{T,2}} \rho^{n_{T,2}}(1-\rho)^{n_{T,1}} \frac{\Gamma(a_\rho+b_\rho)}{\Gamma(a_\rho)\Gamma(b_\rho)} \rho^{a_\rho-1}(1-\rho)^{b_\rho-1} d\rho \right\}, \\ &= \frac{\Gamma(a_\theta+b_\theta)}{\Gamma(a_\theta)\Gamma(b_\theta)} \sum_{n_{T,2}=0}^{n_T} \left\{ \sum_{n_{2,2}\in\mathcal{N}} \left[\binom{n_{T,2}}{n_{2,2}} \frac{\Gamma(a_\theta+n_{2,2})\Gamma(b_\theta+n_{1,2})}{\Gamma(a_\theta+b_\theta+n_{T,2})} \right] \right. \\ &\quad \left. \times \binom{n_T}{n_{T,2}} \frac{\Gamma(a_\rho+b_\rho)}{\Gamma(a_\rho)\Gamma(b_\rho)} \frac{\Gamma(a_\rho+n_{T,2})\Gamma(b_\rho+n_{T,1})}{\Gamma(a_\rho+b_\rho+n_T)} \right\}. \end{aligned}$$

(iii) The assurance for the *sensitivity and specificity* together, based on a sample size of n_T, conditional on $n_{T,1}$ and hence $n_{T,2}$, is

$$\begin{aligned}
A_{\lambda,\theta}(n_T \mid n_{T,1}) &= \int \Pr(\text{Sensitivity and specificity achieved} \mid \lambda, \theta)\pi(\lambda)\pi(\theta)d\lambda d\theta, \\
&= \int \Pr(\text{Sensitivity achieved} \mid \lambda)\pi(\lambda)d\lambda \\
&\quad \times \int \Pr(\text{Specificity achieved} \mid \theta)\pi(\theta)d\theta, \\
&= \frac{\Gamma(a_\lambda + b_\lambda)}{\Gamma(a_\lambda)\Gamma(b_\lambda)} \sum_{n_{1,1}\in\mathcal{N}_1} \binom{n_{T,1}}{n_{1,1}} \frac{\Gamma(a_\lambda + n_{1,1})\Gamma(b_\lambda + n_{2,1})}{\Gamma(a_\lambda + b_\lambda + n_{T,1})} \\
&\quad \times \frac{\Gamma(a_\theta + b_\theta)}{\Gamma(a_\theta)\Gamma(b_\theta)} \sum_{n_{2,2}\in\mathcal{N}_2} \binom{n_{T,2}}{n_{2,2}} \frac{\Gamma(a_\theta + n_{2,2})\Gamma(b_\theta + n_{1,2})}{\Gamma(a_\theta + b_\theta + n_{T,2})},
\end{aligned}$$

from (1) and (2) above, where $\mathcal{N}_1$ contains the values $n_{1,1} \leq c_1$ and $n_{1,1} \geq c_2$ that give a posterior interval narrower than w^*_λ for the sensitivity and $\mathcal{N}_2$ contains the values $n_{2,2} \leq \tilde{c}_1$ and $n_{2,2} \geq \tilde{c}_2$ that give a posterior interval narrower than w^*_θ for the specificity. Thus, the unconditional assurance in this case is

$$\begin{aligned}
A_{\lambda,\theta}(n_T) &= \sum_{n_{T,1}=0}^{n_T} A_{\lambda,\theta}(n_T \mid n_{T,1}) \times f(n_{T,1}), \\
&= \frac{\Gamma(a_\lambda + b_\lambda)}{\Gamma(a_\lambda)\Gamma(b_\lambda)} \frac{\Gamma(a_\theta + b_\theta)}{\Gamma(a_\theta)\Gamma(b_\theta)} \sum_{n_{T,1}=0}^{n_T} \left\{ \sum_{n_{1,1}\in\mathcal{N}_1} \left[\binom{n_{T,1}}{n_{1,1}} \frac{\Gamma(a_\lambda + n_{1,1})\Gamma(b_\lambda + n_{2,1})}{\Gamma(a_\lambda + b_\lambda + n_{T,1})} \right] \right. \\
&\quad \times \sum_{n_{2,2}\in\mathcal{N}_2} \left[\binom{n_{T,2}}{n_{2,2}} \frac{\Gamma(a_\theta + n_{2,2})\Gamma(b_\theta + n_{1,2})}{\Gamma(a_\theta + b_\theta + n_{T,2})} \right] \\
&\quad \left. \times \binom{n_T}{n_{T,1}} \frac{\Gamma(a_\rho + b_\rho)}{\Gamma(a_\rho)\Gamma(b_\rho)} \frac{\Gamma(a_\rho + n_{T,1})\Gamma(b_\rho + n_{T,2})}{\Gamma(a_\rho + b_\rho + n_T)} \right\}.
\end{aligned}$$

B Other considerations for prior specification and model checking

B.1 Design and analysis priors

In practice, there are two prior distributions for each parameter of interest. Consider the sensitivity, for example: it has a prior used for the *design* of the diagnostic accuracy study given in equation (4) of the main paper, and another used for the *analysis* of the diagnostic accuracy study given in equation (1) of the main paper. We have assumed that these are the same, and are based on both expert judgements and the results of the analytical validity stage. This would be the most desirable approach since it utilises the available information at all stages in the development process.

However, the use of expert judgement and/or the results of the analytical validity study could be controversial when inferring the sensitivity and specificity of the index test following the diagnostic accuracy study. In this case, we could use a weaker prior (with $a_\lambda = b_\lambda = 1$, for example) in the analysis, and retain the original prior in the design so that the design and analysis priors differ. This is consistent with the Bayesian approach, as the priors represent the beliefs of different individuals. For a discussion on this, see O'Hagan et al. [2005], Wilson and Farrow [2021].

In Section B.4 below, we will consider the use of flat analysis priors for the sensitivity and/or prevalence in the VAP study, and the effect this has on the sample size and resulting inference.

B.2 Alternative uses of the analytical validity data

Utilising data from the analytical validity stage in the way suggested in the main paper makes the assumption that the observations in the two stages — analytical validity and diagnostic accuracy — are exchangeable. That is, they form random samples from the sample population of interest with the same sensitivity, specificity and prevalence. This may be a strong assumption in some cases.

A weaker assumption would be to assume that the sensitivities, specificities and prevalences of the test are positively correlated at the two stages. Setting up a prior based on this assumption allows us to "borrow strength", still utilising the data from the analytical validity study in the diagnostic accuracy study, but not requiring the strong assumption of exchangeability.

To achieve this, we could use a *hierarchical prior*. Suppose that the prior for the sensitivity before the analytical validity study is $\lambda^0 \sim \text{Beta}(a_\lambda^0, b_\lambda^0)$ and before the diagnostic accuracy study is $\lambda^1 \sim \text{Beta}(a_\lambda^1, b_\lambda^1)$. The parameters of these beta distributions are then given prior distributions:

$$\begin{aligned} a_\lambda^j &\sim \text{Gamma}(r_a, k_a), \\ b_\lambda^j &\sim \text{Gamma}(r_b, k_b), \quad j = \{0, 1\}, \end{aligned}$$

and the hyper-parameters are given suitable prior distributions, such as:

$$\begin{aligned} r_a \sim \text{Gamma}(2, 2), &\quad k_a \sim \text{Gamma}(2, 2), \\ r_b \sim \text{Gamma}(2, 2), &\quad k_b \sim \text{Gamma}(2, 2). \end{aligned}$$

The use of common parameters r_a, k_a, r_b, k_b across the two stages induces the correlations. The same structure could also be used for the specificity and prevalence. Inference could then be carried out using Markov Chain Monte Carlo techniques [Gelman et al., 2013].

Other options would be to use a mixture prior, such as a meta analytic predictive prior [Schmidli et al., 2014], or to informally use the historical data in an evidence dossier as part of a formal expert elicitation process [O'Hagan et al., 2006].

B.3 Assessing the prior sensitivity in the VAP study

To assess the effect that small changes to the priors have on the resulting sample size required for the VAP diagnostic accuracy study (from Section 5 in the main paper), we carry out a sensitivity analysis.

Following the approach outlined in Section 5.2 of the main paper, we conduct a grid search for the sensitivity and prevalence priors, with the combinations of parameter values represented by the black curves and the original parameter values by the red dots in Figure 1.

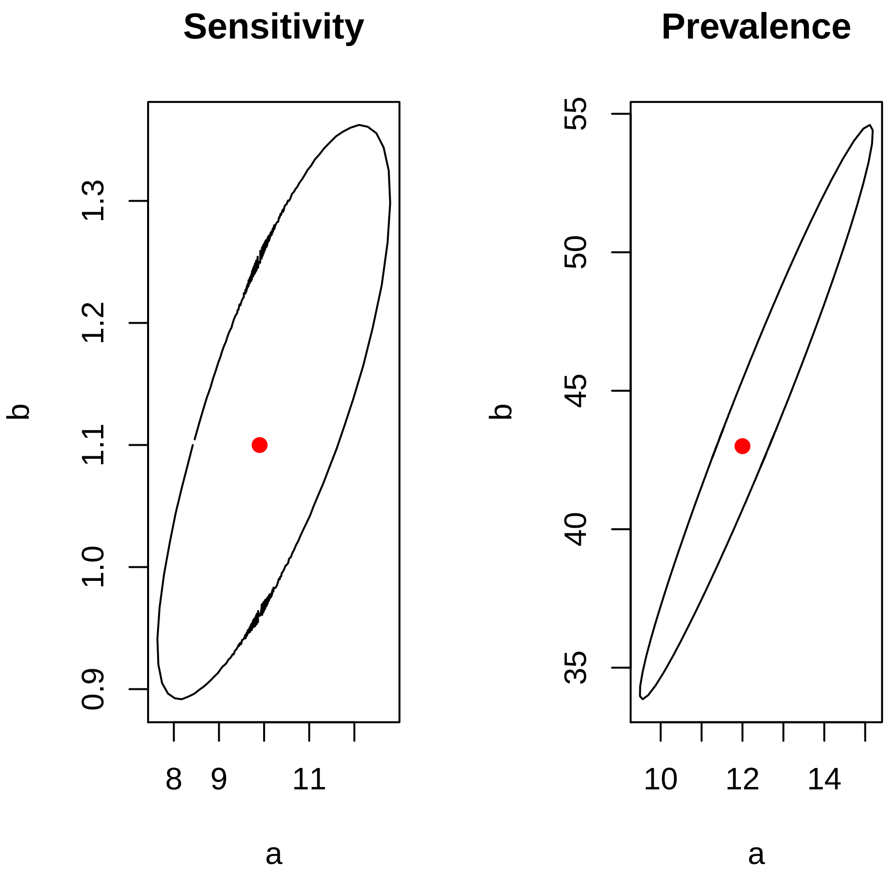

Figure 1: The combinations of beta distribution parameters (a, b) considered in the prior sensitivity analysis (black curves), and the original prior parameter values (red dots) for the sensitivity of the test (left) and the prevalence of VAP (right).

The prior parameter values which we consider in the sensitivity analysis form ellipses around their original values, as a result of using the Hellinger distance. That is, we search beta distributions which are a particular distance (in this case, $\epsilon = 0.00354$) from the original beta prior distribution, rather than searching individual prior parameters which are a particular distance from their original values. The resulting effects on the assurance and sample size are provided in Table 2 of the main paper.

B.4 Alternative uses of assurance

In the VAP study, used as a real-life application in the main paper, it seemed reasonable to assume that the patients in the diagnostic accuracy study were exchangeable with those in the single centre

observational study. Therefore, we included the observations from the observational study in the prior for the diagnostic accuracy study, both in its design and its analysis. However, since the exchangeability assumption may not always hold (as discussed in Section B.2), we explore some alternative uses of assurance in this context.

The first — referred to as the 'combination' approach — uses data from the biomarker selection study to form the design priors, but flat analysis priors (see Section 5.1 in the main paper). That is, $\lambda \sim \text{Beta}(25.9, 2.1)$ and $\rho \sim \text{Beta}(29, 98)$ for the design priors, and $\lambda \sim \text{Beta}(1, 1)$ and $\rho \sim \text{Beta}(1, 1)$ for the analysis priors. The others are 'hybrid' approaches, combining an informative analysis prior for one of the parameters with a non-informative prior for the other. In order to use all of the available information to choose the sample size, we use informative design priors for each approach. The results of using these alternative methods, together with the original 'standard' approach, are summarised in Table 1.

Approach	Design priors	Analysis priors	Sample size	Estimates
Standard	$\lambda \sim \text{Beta}(25.9, 2.1)$	$\lambda \sim \text{Beta}(25.9, 2.1)$	106	$\hat{\lambda} = 0.949$
	$\rho \sim \text{Beta}(29, 98)$	$\rho \sim \text{Beta}(29, 98)$		$\hat{\rho} = 0.296$
Combination	$\lambda \sim \text{Beta}(25.9, 2.1)$	$\lambda \sim \text{Beta}(1, 1)$	290	$\hat{\lambda} = 0.945$
	$\rho \sim \text{Beta}(29, 98)$	$\rho \sim \text{Beta}(1, 1)$		$\hat{\rho} = 0.355$
Hybrid I	$\lambda \sim \text{Beta}(25.9, 2.1)$	$\lambda \sim \text{Beta}(25.9, 2.1)$	106	$\hat{\lambda} = 0.949$
	$\rho \sim \text{Beta}(29, 98)$	$\rho \sim \text{Beta}(1, 1)$		$\hat{\rho} = 0.355$
Hybrid II	$\lambda \sim \text{Beta}(25.9, 2.1)$	$\lambda \sim \text{Beta}(1, 1)$	290	$\hat{\lambda} = 0.945$
	$\rho \sim \text{Beta}(29, 98)$	$\rho \sim \text{Beta}(29, 98)$		$\hat{\rho} = 0.296$

Table 1: A comparison of four approaches to sample size determination using assurance. The parameter estimates provided are the posterior means for the parameters.

Using non-informative analysis priors for both parameters results in a sample size of 290; much larger than the 106 we originally found using the'standard' approach. This illustrates the utility of including all available information in the parameter estimates. Similarly, when a non-informative analysis prior is only used on the sensitivity in the 'Hybrid II' approach, the sample size remains large. In contrast, when a non-informative prior is used only on the prevalence in the 'Hybrid I' approach, the original sample size is recovered. This is because the sample size calculation is based on the interval estimate for the sensitivity and not the prevalence (and the design prior on the prevalence has not changed). In this case, however, the posterior mean for the prevalence increases.

C Additional sample size comparisons

The tables in this section provide additional analysis for Section 6.1 of the main paper, which compares sample sizes for the BAM with a number of other methods. Full details of the comparisons, including the notation used below, are provided there.

Prev.	Sens.	Wald	CP	AC	BAM 25	BAM 50	BAM 75	Non-inf
0.90	0.70	80	88	77	56	28	1	79
	0.75	75	82	70	50	20	1	73
	0.80	66	74	63	41	9	1	66
	0.85	55	64	55	28	1	1	56
0.70	0.70	103	113	99	82	53	24	103
	0.75	96	105	90	74	43	12	96
	0.80	85	95	80	62	30	2	86
	0.85	70	82	70	48	12	2	74
0.50	0.70	144	158	138	132	99	68	149
	0.75	134	146	126	120	86	55	138
	0.80	118	132	112	104	69	36	124
	0.85	98	114	98	83	46	13	106
0.30	0.70	240	264	230	256	214	179	259
	0.75	224	244	210	235	191	157	240
	0.80	197	220	187	207	164	126	215
	0.85	164	190	164	171	125	88	185

Table 2: Sample sizes required based on different prevalences and sensitivities, $\alpha = 0.05, \beta = 0.8$ and $w^* = 0.22$. CP stands for Clopper-Pearson, AC stands for Agresti-Coull, Non-inf is assurance using a non-informative analysis prior and BAM 25, 50 and 75 are assurance using prior sample sizes of 25, 50 and 75 respectively.

Prev.	Sens.	Wald	CP	AC	BAM 25	BAM 50	BAM 75	Non-inf
0.90	0.70	118	127	116	97	68	40	119
	0.75	109	118	106	88	57	28	110
	0.80	96	104	94	75	43	13	98
	0.85	79	89	80	57	24	1	83
	0.90	60	70	67	35	1	1	65
0.70	0.70	152	163	149	136	105	77	156
	0.75	140	152	136	125	92	62	144
	0.80	123	135	122	108	74	43	128
	0.85	102	115	103	86	50	17	109
	0.90	78	90	86	57	19	2	85
0.50	0.70	212	228	208	211	175	144	224
	0.75	196	212	190	194	157	124	207
	0.80	172	188	170	171	131	98	184
	0.85	142	160	144	139	99	64	157
	0.90	108	126	120	97	56	23	123
0.30	0.70	354	380	347	399	348	310	390
	0.75	327	354	317	367	316	277	360
	0.80	287	314	284	326	275	234	322
	0.85	237	267	240	272	220	178	274
	0.90	180	210	200	199	149	108	215

Table 3: Sample sizes required based on different prevalences and sensitivities, $\alpha = 0.05, \beta = 0.8$ and $w^* = 0.18$. CP stands for Clopper-Pearson, AC stands for Agresti-Coull, Non-inf is assurance using a non-informative analysis prior and BAM 25, 50 and 75 are assurance using prior sample sizes of 25, 50 and 75 respectively.

Prev.	Sens.	Wald	CP	AC	BAM 25	BAM 50	BAM 75	Non-inf
0.90	0.70	194	206	190	178	147	119	197
	0.75	176	188	172	164	131	101	182
	0.80	155	167	153	143	108	77	161
	0.85	127	140	130	115	79	47	136
	0.90	97	109	100	79	42	1	104
0.70	0.70	249	265	245	243	210	180	258
	0.75	226	242	220	224	188	157	238
	0.80	199	215	196	198	159	127	211
	0.85	163	180	168	163	122	88	178
	0.90	125	140	129	115	75	41	137
0.50	0.70	348	370	342	368	326	292	372
	0.75	316	338	308	340	296	260	343
	0.80	278	300	274	302	256	218	305
	0.85	228	252	234	251	203	166	257
	0.90	174	196	180	183	136	99	198
0.30	0.70	580	617	570	680	614	569	648
	0.75	527	564	514	629	562	515	598
	0.80	464	500	457	561	495	445	534
	0.85	380	420	390	473	406	357	452
	0.90	290	327	300	352	291	244	349

Table 4: Sample sizes required based on different prevalences and sensitivities, $\alpha = 0.05, \beta = 0.8$ and $w^* = 0.14$. CP stands for Clopper-Pearson, AC stands for Agresti-Coull, Non-inf is assurance using a non-informative analysis prior and BAM 25, 50 and 75 are assurance using prior sample sizes of 25, 50 and 75 respectively.

D Additional interval width comparisons

The figures in this section provide additional analysis for Section 6.2 of the main paper, which compares the widths of interval estimates for the BAM with a number of other methods. Full details of the comparisons, including the notation used below, are provided there.

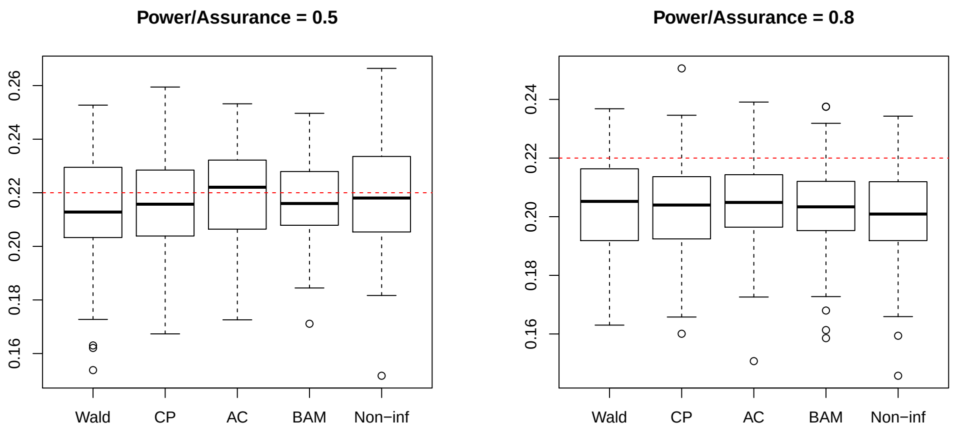

Figure 2: The width of 95% confidence or posterior probability intervals based on $M = 100$ simulations for the Normal approximation (Wald), Clopper-Pearson (CP), Agresti-Coull (AC), Assurance (BAM) and Assurance using a non-informative analysis prior (Non-inf)). The power/assurance used to choose the sample size was 0.5 (left) and 0.8 (right). The horizontal line is at the desired width of $w^* = 0.22$.

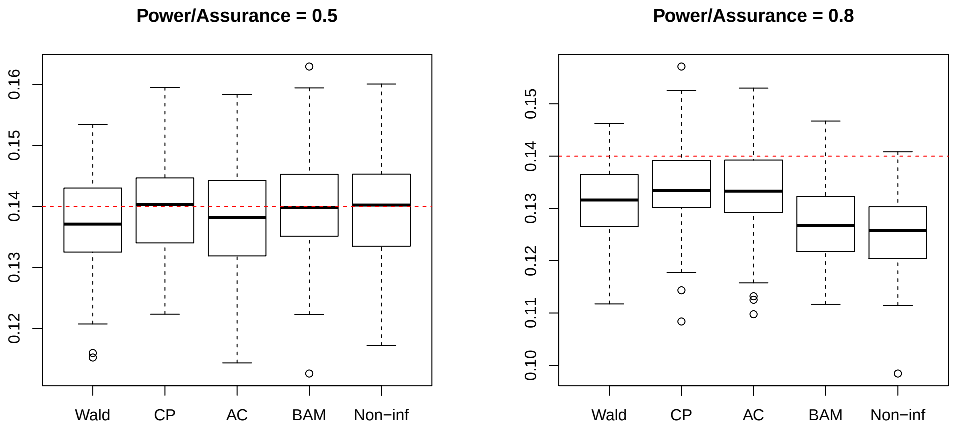

Figure 3: The width of 95% confidence or posterior probability intervals based on $M = 100$ simulations for Wald, CP, AC, BAM and Non-inf. The power/assurance used to choose the sample size was 0.5 (left) and 0.8 (right). The horizontal line is at the desired width of $w^* = 0.14$.

E Investigation of properties compared to the "standard approach"

In this section, we provide a comparison (equivalent to those in Sections 6.1 and 6.2 of the main paper) using the "standard approach" to sample size calculations for the frequentist approaches, i.e. via the appropriate sample size formula (if available), or in-built functions within statistical software (e.g. the `binDesign` function from the `binGroup` R package).

E.1 Sample size comparisons

The results for the "standard approach" are provided in Figure 4.

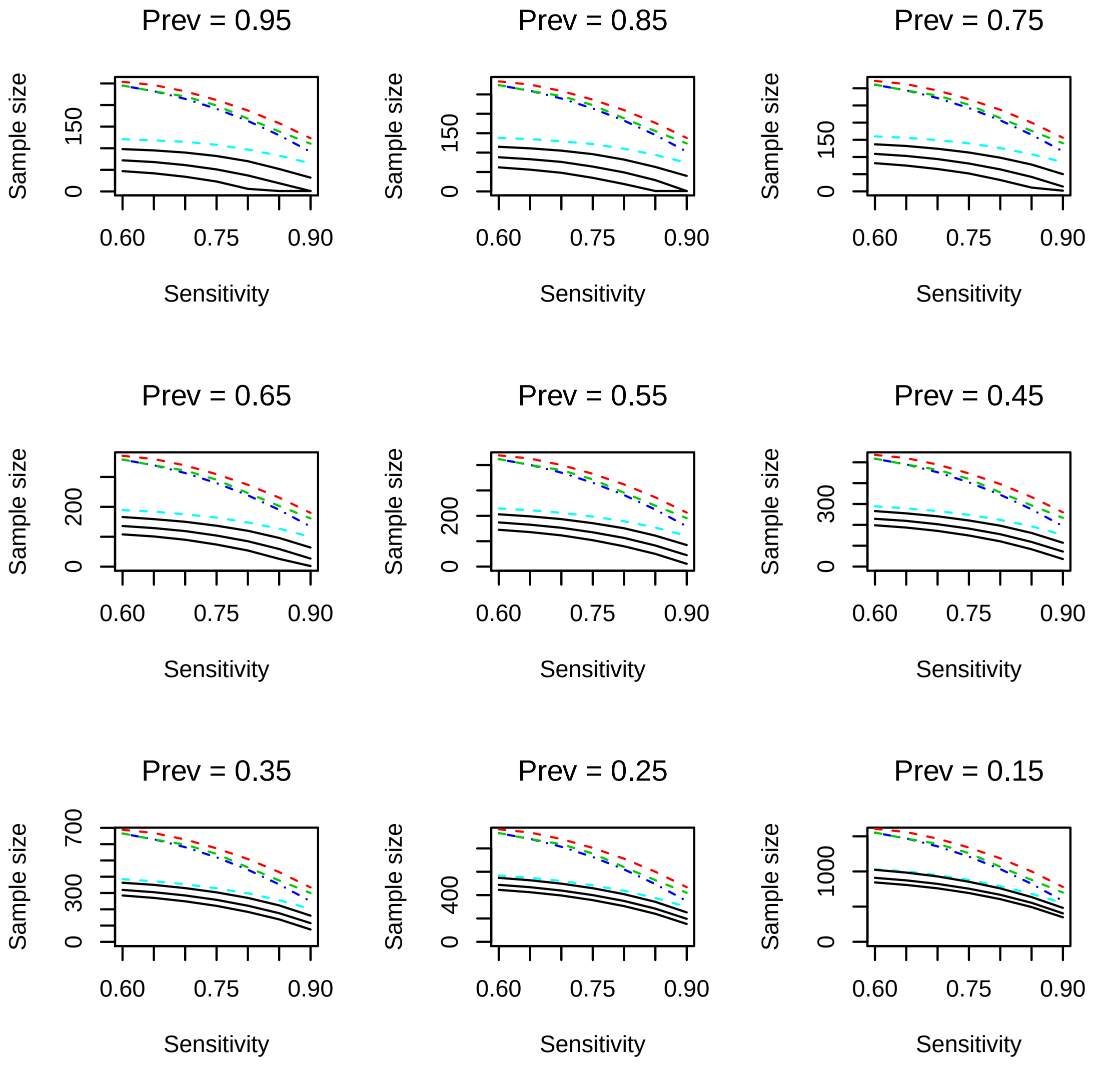

Figure 4: A comparison of the sample sizes required based on power calculations using a normal approximation (dark blue), Clopper-Pearson (red), Agresti-Coull (green), assurance (black) and assurance based on non-informative analysis priors (light blue). In each plot there are three black curves corresponding to prior sample sizes of (from top to bottom) 25, 50 and 75.

We see a similar pattern in each plot. The Clopper-Pearson approach always results in the largest sample size, with Normal approximation and Agresti-Coull approach giving similar, slightly smaller sample sizes. For all combinations of sensitivity and prevalence the methods based on power calculations result in larger sample sizes than those based on assurance. The approach based on a flat analysis prior gives a larger sample size than that based on an informative prior. As the prior sample size increases, the required sample size in the diagnostic accuracy study decreases.

Thus, even if a flat prior will be used in the analysis of the diagnostic accuracy study, assurance can reduce the required sample size by utilising previously collected data in the design prior. The comparisons were repeated for all of the combinations of parameters in Web Appendix C. The main message is consistent across all of the parameter combinations considered: assurance reduces the required sample size for all of the considered prior sample sizes, including when a non-informative analysis prior is used.

E.2 Interval width comparisons

The results for the "standard approach" are provided in Figure 5 for a power/assurance of 0.5 (left) and 0.8 (right). In all cases $\alpha = 0.05$ and $w^* = 0.18$.

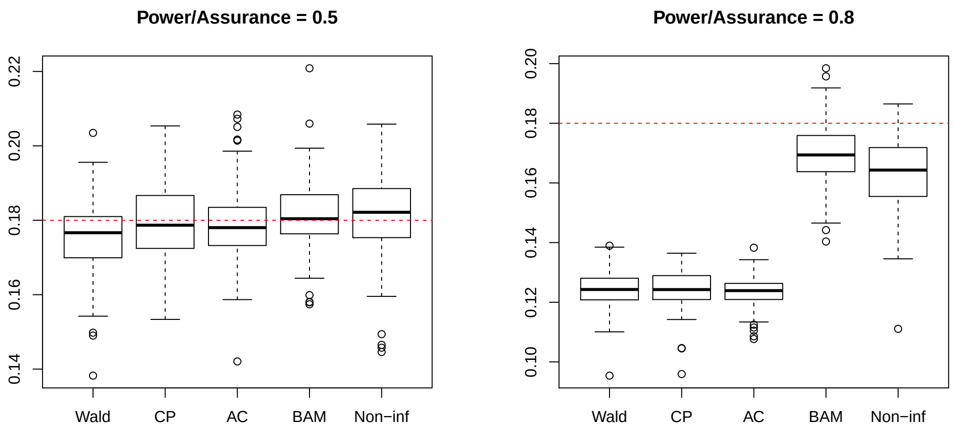

Figure 5: The width of 95% confidence or posterior probability intervals based on $M = 100$ simulations for the Normal approximation, Clopper-Pearson, Agresti-Coull, the BAM and the BAM using a non-informative analysis prior. The power/assurance used to choose the sample size was 0.5 (left) and 0.8 (right). The horizontal line is at the desired width of $w^* = 0.18$.

We see that, when $\beta = 0.5$, the approaches all produce intervals with a similar distribution of widths. Therefore, the sample size reduction observed in the previous section in the assurance-based approaches does not come at the expense of less precision in inference. Moreover, all of the distributions are approximately centred at the target width of $w^* = 0.18$.

When $\beta = 0.8$, however, the assurance-based approaches provide intervals which are both wider on average and have a larger spread of widths than the three power-based approaches. In fact, we see that the power-based approaches produce intervals which are "too narrow", as they are always much narrower than the desired interval width of 0.18. That is, the sample sizes found using these methods could be reduced and still produce suitable intervals. In contrast, the intervals resulting from the assurance-based approaches contain the desired width of 0.18 in the upper tails of their distributions, as would be expected with $\beta = 0.8$.

The simulations were repeated with interval widths of $w^* = 0.22$ and $w^* = 0.14$. The main conclusions remain: for a power/assurance of 0.5 all of the distributions were approximately centred on

the target width but for a power/assurance of 0.8 the power-based methods produced intervals which were consistently too narrow. This suggests that the larger sample sizes produced by the power-based approaches may be because they are producing intervals whose power is too high.

F Properties of the BAM when assuring sensitivity and specificity together

First we consider the required sample size when assuring the sensitivity and specificity together. We follow the same general approach as Section 6.1 of the paper. We suppose that $\alpha = 0.05$, we want the assurance of $\beta = 0.8$ to estimate sensitivity and specificity to within interval widths of first $w^* = 0.18$ and then $w^* = 0.22$ in two-sided intervals. We vary the true sensitivity in the interval $[0.8, 0.95]$, suppose that the true population prevalence is 0.3 and consider an analytic validity sample size of $n = 50$.

In Figure 6 we plot the sensitivity against the required sample size for target interval widths on both sensitivity and specificity of 0.18 (left) and 0.22 (right). The colours of the lines represent a true specificity of 0.8 (black), 0.85 (red), 0.9 (green) and 0.95 (blue).

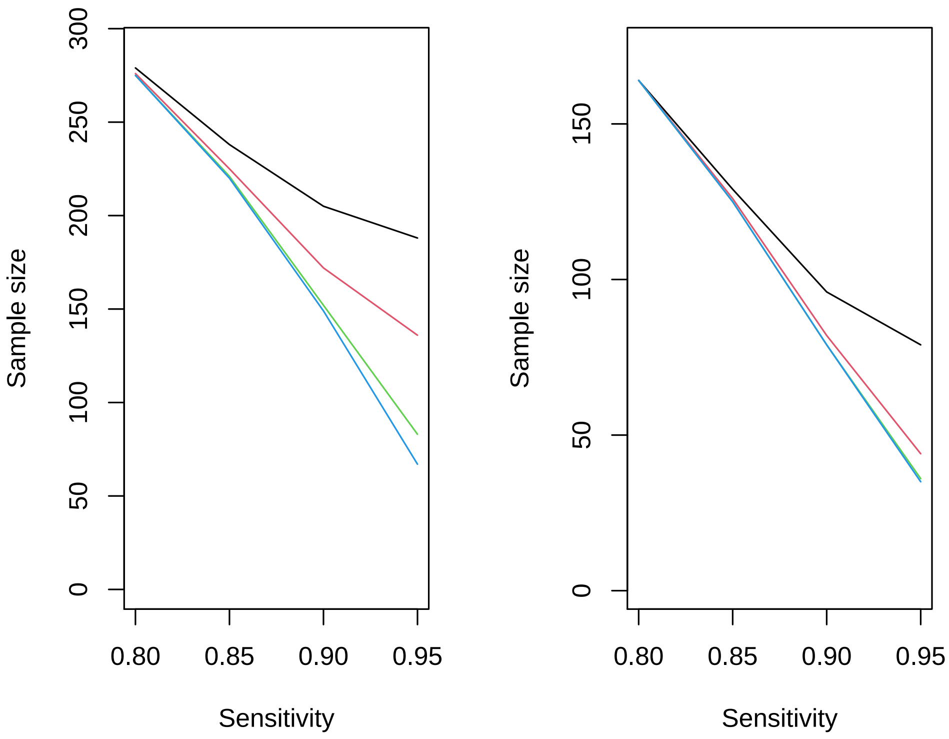

Figure 6: Sensitivity plotted against required sample size when assuring *both* sensitivity and specificity. The colours of the lines represent a specificity of 0.8 (black), 0.85 (red), 0.9 (green) and 0.95 (blue). Target interval widths are 0.18 (left) and 0.22 (right).

We see in both plots that, with a prevalence well below 0.5, the sensitivity dominates the required sample size. For sensitivities around 0.8 the value of the specificity has little effect on the required sample size. As the sensitivity increases, and achieving the required width on the interval for the sensitivity requires fewer participants, the specificity of the test has a stronger influence on the required sample size.

Next we consider the resulting widths of the intervals for sensitivity and specificity. We use the same approach as Section 6.2 of the paper. We consider the widths of the posterior intervals for the sensitivity

and specificity based on 100 simulations. The results are given in Figure 7 for an assurance of 0.5 (left) and 0.8 (right).

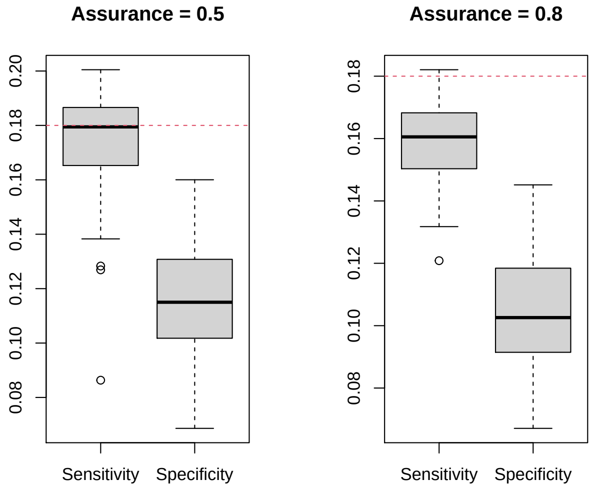

Figure 7: Boxplots of the widths of the posterior intervals for sensitivity and specificity, based on 100 samples, for an assurance of 0.5 (left) and 0.8 (right).

From the boxplots we see that the interval for specificity is always below the target width, whether the assurance is 0.5 or 0.8. Thus the assurance, and hence the sample size required, is based on the interval for the sensitivity. This is a result of the choice of a prevalence of less than 0.5 (and the equal underlying sensitivity and specificity). When the assurance is 0.5, the target width of 0.18 lies almost exactly on the median width for the sensitivity interval, and when the assurance is 0.8, it is in the upper quantiles of the distribution of interval widths for the sensitivity.

References

Andrew Gelman, John B. Carlin, Hal S. Stern, and Donald B. Rubin. *Bayesian Data Analysis*. Chapman and Hall/CRC, 3rd ed. edition, 2013.

A. O'Hagan, J.W. Stevens, and M.J. Campbell. Assurance in clinical trial design. *Pharmaceutical Statistics*, 4:187–201, 2005.

Anthony O'Hagan, Caitlin E Buck, Alireza Daneshkhah, J Richard Eiser, Paul H Garthwaite, David J Jenkinson, Jeremy E Oakley, and Tim Rakow. *Uncertain judgements: eliciting experts' probabilities*. John Wiley & Sons, 2006.

Heinz Schmidli, Sandro Gsteiger, Satrajit Roychoudhury, Anthony O'Hagan, David Spiegelhalter, and Beat Neuenschwander. Robust meta-analytic-predictive priors in clinical trials with historical control information. *Biometrics*, 70(4):1023–1032, 2014.

K.J. Wilson and M. Farrow. Assurance for sample size determination in reliability demonstration testing. *Technometrics*, 2021. doi: 10.1080/00401706.2020.1867646.
